# The role of relative fat mass in gallstone risk assessment: findings from the NHANES 2017–2020 survey

**DOI:** 10.3389/fnut.2025.1575524

**Published:** 2025-04-30

**Authors:** Li Wang, Shan Cao, Guodong Song

**Affiliations:** ^1^Department of Gastrointestinal Surgery, The Second Hospital of Tianjin Medical University, Tianjin, China; ^2^Department of Respiratory, The Second Hospital of Tianjin Medical University, Tianjin, China

**Keywords:** gallstones, relative fat mass, NHANES, cross-sectional study, risk factor

## Abstract

**Background:**

Gallstones are a prevalent condition that can lead to significant morbidity and healthcare costs. Relative fat mass (RFM), as a potential marker of body fat distribution, may offer insights beyond traditional metrics like body mass index (BMI) and waist circumference. This study aims to investigate the association between RFM and gallstone prevalence in the U.S. population.

**Methods:**

The study cohort comprised 6,881 participants obtained from the National Health and Nutrition Examination Survey (NHANES) conducted between 2017 and 2020. Participants were stratified into quartiles (Q1–Q4) based on their RFM. To evaluate the associations, multivariable logistic regression analyses were employed to assess odds ratios (OR) for gallstone risk across different quartiles of RFM. Additionally, restricted cubic spline analysis was conducted to ascertain the relationship trend while subgroup analyses examined interactions based on age, sex, race, education level, and lifestyle factors.

**Results:**

The analysis revealed significant associations for participants within the higher RFM quartiles (Q3 and Q4), with ORs of 2.58 (95% CI: 1.65, 4.04) and 6.30 (95% CI: 3.63, 10.93), respectively, compared to Q1. The findings consistently indicated that RFM, particularly in Q4, is a strong predictor of gallstone risk, demonstrating superior predictive performance relative to waist circumference and BMI, as evidenced by an AUC of 0.702.

**Conclusion:**

Elevated RFM is a noteworthy predictor of gallstone risk in the studied population, suggesting its potential utility in clinical risk assessment frameworks. Future research should focus on elucidating the underlying mechanisms driving this association and exploring RFM's applicability as a pragmatic tool in clinical practice for gallstone risk stratification.

## Introduction

Gallstones are a prevalent biliary tract disorder worldwide, affecting roughly 10–15% of the global population, with incidence rates differing across countries (1–3). These conditions are associated with significant health complications, including cholecystitis, cholangitis, pancreatitis, and biliary obstruction, which impose a considerable burden on both patients and healthcare systems (4, 5). The development of gallstones is influenced by numerous environmental and genetic factors (6, 7). Identifying risk factors for gallstone formation and developing predictive tools is essential for early intervention and prevention. Obesity has long been recognized as a major risk factor for gallstones, with traditional measures such as body mass index (BMI) and waist circumference (WC) commonly used in clinical and epidemiological studies (8, 9). However, these metrics have limitations in accurately reflecting body fat distribution and metabolic health, highlighting the need for more precise indicators.

Relative fat mass (RFM), a novel anthropometric index calculated using height and WC, has emerged as a promising tool for assessing adiposity and its associated health risks (10, 11). Unlike BMI and WC, RFM offers a more accurate estimation of body fat percentage and distribution, making it potentially more effective in predicting obesity-related conditions, including gallstones (12, 13). Increasing evidence suggests that RFM is linked to various metabolic diseases, including hypertension, type 2 diabetes, and cardiovascular conditions (14–16). However, the association between RFM and gallstones remains underexplored. Furthermore, existing research often relies on regional or small-scale datasets, which restrict the generalizability of the findings. Consequently, it is imperative to explore the association between RFM and gallstones utilizing nationally representative data to improve our understanding of this connection.

In this investigation, data from the National Health and Nutrition Examination Survey (NHANES) spanning the years 2017 to 2020 will be analyzed to assess the relationship between RFM and the prevalence of gallstones. Utilizing multivariable logistic regression models and restricted cubic spline (RCS) analysis, this study aims to evaluate the predictive capability of RFM concerning gallstone risk and to analyze potential interactions with demographic and lifestyle factors. The findings will provide valuable insights into the role of RFM as a clinical tool for gallstone risk assessment and contribute to the development of targeted interventions aimed at reducing gallstone prevalence through enhanced obesity management.

## Materials and methods

### Study design

**Figure 1:**
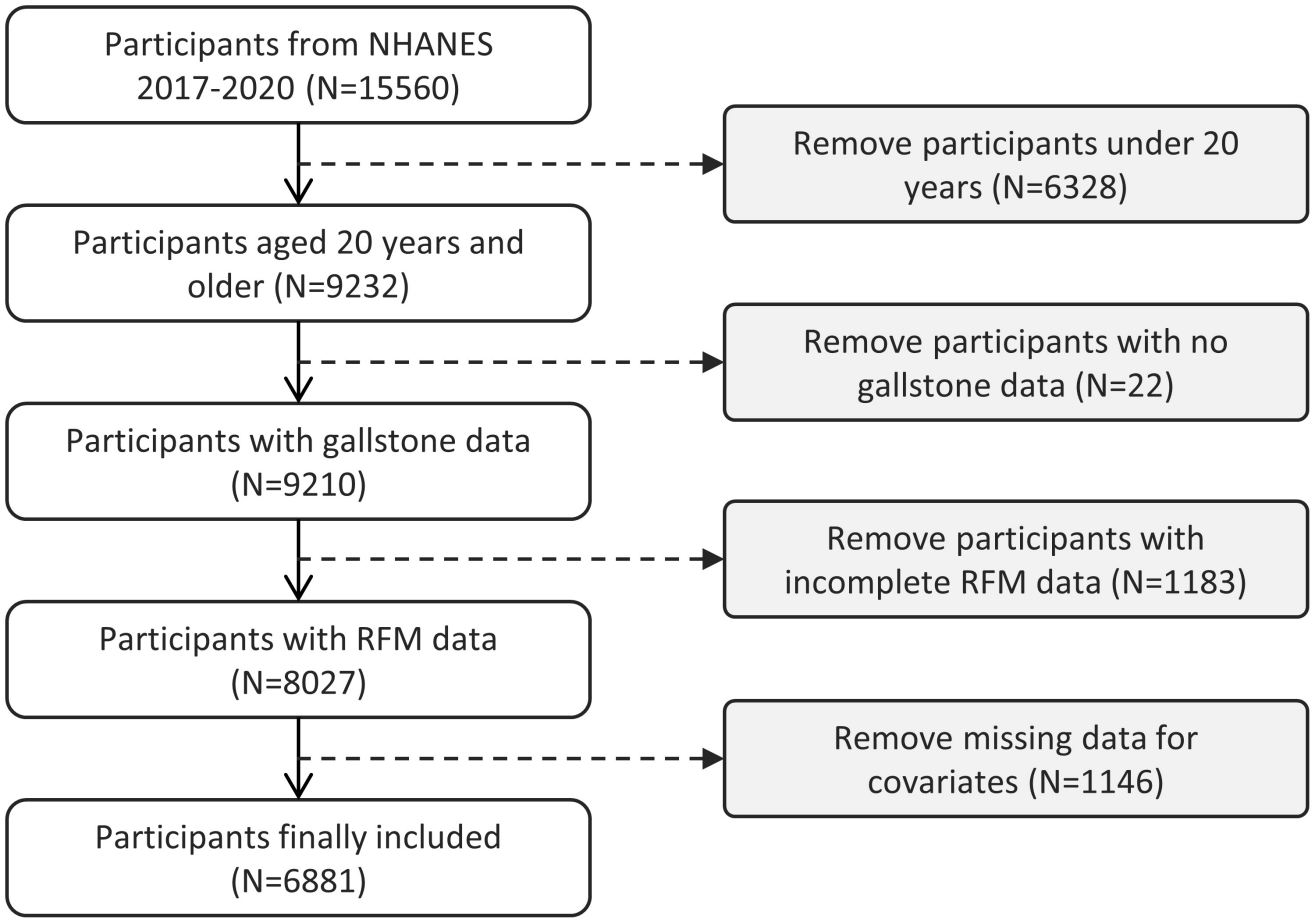
Flowchart of participant selection in NHANES 2017–2020.

The NHANES, managed by the Centers for Disease Control and Prevention (CDC), is a cross-sectional study designed to evaluate the health, nutrition, and epidemiological characteristics of both adults and children in the United States. The survey integrates data from health interviews and physical exams, using a multi-stage, stratified random sampling approach to ensure a representative sample of the U.S. population. This methodology allows for comprehensive assessment of the nation's health and nutritional status. All participants voluntarily enrolled in the study and provided signed informed consent. This study utilized data from the years 2017 to 2020, encompassing a total of 15,560 participants. Participants were eligible for inclusion if they were aged 20 years or older, had complete gallstone-related data, full RFM data, and relevant covariate data. The exclusion criteria were defined as follows: (1) individuals younger than 20 years of age; (2) individuals with missing information regarding gallstone disease; (3) participants with incomplete data on RFM; (4) subjects lacking data on covariates. The detailed screening process is illustrated in Figure 1. Ultimately, 6,881 eligible participants were included in the analysis, of whom 712 had been diagnosed with gallstones.

### Study variables

#### Definition of gallstones

The presence of gallstones was the primary outcome variable in this study. Gallstone presence was determined by the survey question, “Has a doctor ever told you that you have gallstones?” The responses were classified based on participants' answers.

#### Definition of RFM

Height and WC were measured by trained health professionals at the Mobile Examination Center (MEC), and the RFM was calculated based on the participant's gender. Participants stood barefoot in the MEC with their backs against a specialized height-measuring device, ensuring their heads were horizontally aligned with their backs. WC was measured above the iliac crest, at the mid-axillary line. Both height and WC were recorded in centimeters with a precision of 0.1 cm (17). The formula for calculating RFM is as follows: RFM = 64 – (20 × height/WC) + (12 × gender), where height and WC are measured in centimeters, with females coded as 1 and males as 0(10). Participants were classified into four groups based on the quartiles of RFM: Q1, Q2, Q3 and Q4.

### Covariates

This study incorporated age, gender, race, education level, and marital status as covariates, based on prior research (18, 19). Participants were classified into two age groups: those under 60 years and those aged 60 years or older. Race and ethnicity were categorized into distinct groups: Mexican African American, other Hispanic, non-Hispanic White, non-Hispanic Black, and various other races (including individuals identifying as multiracial). Education levels were categorized into three groups: low (<9th grade, 9th to 11th grade, and those without a high school diploma); moderate (high school graduate/GED, some college, or associate degree); and high (college graduate and above). Marital status was categorized into three groups: cohabitation (married/living with a partner), unmarried, and living alone (widowed/divorced/separated). Hypertension and hypercholesterolemia were identified based on participants' self-reports (presence or absence). Diabetes status was categorized based on the response to “Has a doctor told you that you have diabetes?” into three groups: yes, no, and borderline (blood sugar is higher than normal but not high enough to be called diabetes). Smoking status was categorized based on the question “Have you smoked more than 100 cigarettes?” (SMQ020), with “yes” indicating a smoker and “no” indicating a non-smoker. Alcohol consumption was categorized based on “frequency of alcohol consumption in the past 12 months” (ALQ121) into three groups: low-frequency drinkers (never drank in the past year, 1–2 times, 3–6 times, 7–11 times); moderate-frequency drinkers (1 time per month, 2–3 times per month, 1 time per week, 2 times per week); and high-frequency drinkers (3–4 times per week, almost every day, every day). Missing covariate data were excluded from the analysis.

### Statistical methods

Statistical analyses were performed following the NHANES guidelines, taking into account both the complex sampling design and the application of sampling weights. Continuous variables are presented as means ± standard deviations (SD), while categorical variables are reported as frequencies or percentages. Three multivariable logistic regression models were employed to assess the relationship between RFM and gallstone risk. Model 1 did not adjust for covariates, Model 2 adjusted for gender, age, race, education level, and marital status, while Model 3 additionally adjusted for hypertension, diabetes, hypercholesterolemia, smoking status, and alcohol consumption, relative to Model 2. RCS analysis was employed to further investigate the nonlinear relationship between RFM and gallstones. The placement of knots in the restricted cubic spline analysis was based on the default settings in the statistical software, with knots placed at the 25th, 50th, and 75th percentiles of the distribution of RFM values. Then, we conducted sensitivity analyses with alternative knot placements, and the results were consistent with our original findings. Additionally, subgroup analyses were performed to evaluate the predictive role of RFM across various groups. To compare the predictive performance of the different metrics (RFM, WC, and BMI), we used Receiver Operating Characteristic (ROC) curves and the corresponding Area Under the Curve (AUC). The ROC curves were generated by plotting the true positive rate against the false positive rate for each model. All statistical analyses were conducted using R version 4.3.2, with a *P* < 0.05 considered statistically significant.

## Results

### Baseline characteristics of study samples

Table 1 presents the basic demographic characteristics of the study participants. A total of 6,881 participants were included in this study, of which 712 had gallstones and 6,169 did not, resulting in a gallstone prevalence of 10.3%. In the comparison between the two groups, the gallstone group exhibited a higher RFM (41.6 ± 8.15), which was significantly higher than the non-gallstone group (35.3 ± 8.69), with a statistically significant difference (*P* < 0.001). Additionally, the gallstone group was characterized by an older age and a higher prevalence among females. Non-Hispanic whites, individuals with moderate education levels, and those living alone were more likely to have gallstones. Further analysis indicated that individuals with comorbid hypertension, diabetes, hypercholesterolemia, smoking habits, and low-frequency alcohol consumption had a higher probability of developing gallstones.

**Table 1:** Baseline data summary for NHANES study participants (*N* = 6.881).

**Characteristics**	**Without gallstone disease (*N* = 6,169, 89.7%)**	**With gallstone disease (*N* = 712, 10.3%)**	***P* value**
Age (years)	49.6 (17.3)	57.4 (15.6)	<0.001
**Age category**			<0.001
<60 years	4,089 (66.3%)	358 (50.3%)	
≥60 years	2,080 (33.7%)	354 (49.7%)	
**Gender**			<0.001
Male	3,304 (53.6%)	212 (29.8%)	
Female	2,865 (46.4%)	500 (70.2%)	
**Race**			<0.001
Mexican American	719 (11.7%)	93 (13.1%)	
Non-Hispanic Black	1,688 (27.4%)	149 (20.9%)	
Non-Hispanic White	2,233 (36.2%)	318 (44.7%)	
Other Hispanic	618 (10.0%)	79 (11.1%)	
Other Race	911 (14.8%)	73 (10.3%)	
**Education category**			0.005
Low	1,009 (16.4%)	114 (16.0%)	
Moderate	3,586 (58.1%)	454 (63.8%)	
High	1,574 (25.5%)	144 (20.2%)	
**Marital**			<0.001
Cohabitation	3,572 (57.9%)	428 (60.1%)	
Never married	1,258 (20.4%)	93 (13.1%)	
Living alone	1,339 (21.7%)	191 (26.8%)	
**Hypertension**			<0.001
No	3,933 (63.8%)	327 (45.9%)	
Yes	2,236 (36.2%)	385 (54.1%)	
**Diabetes**			<0.001
Borderline	181 (2.93%)	19 (2.67%)	
No	5,154 (83.5%)	518 (72.8%)	
Yes	834 (13.5%)	175 (24.6%)	
**Cholesterol**			<0.001
No	4,030 (65.3%)	355 (49.9%)	
Yes	2,139 (34.7%)	357 (50.1%)	
**Smoke**			0.009
No	3,399 (55.1%)	355 (49.9%)	
Yes	2,770 (44.9%)	357 (50.1%)	
**Alcohol group**			<0.001
Low frequency	2,910 (47.2%)	432 (60.7%)	
Moderate frequency	1,821 (29.5%)	179 (25.1%)	
High frequency	1,438 (23.3%)	101 (14.2%)	
BMI (Kg/m^2^)	29.7 (7.14)	33.6 (8.58)	<0.001
**BMI category**			<0.001
<25.0	1,636 (26.5%)	76 (10.7%)	
25.0–29.9	1,975 (32.0%)	196 (27.5%)	
≥29.9	2,558 (41.5%)	440 (61.8%)	
RFM	35.3 (8.69)	41.6 (8.15)	<0.001
**RFM category**			<0.001
Q1 (≤29.5)	1,657 (26.9%)	63 (8.85%)	
Q2 (29.5–35.2)	1,595 (25.9%)	125 (17.6%)	
Q3 (35.2–43.3)	1,566 (25.4%)	154 (21.6%)	
Q4 (>43.3)	1,351 (21.9%)	370 (52.0%)	

### Higher RFM scores associated with increased gallstone incidence

Multivariate regression analysis was conducted to explore the relationship between RFM and gallstone prevalence while controlling for various confounding factors (Table 2). In Model 1, compared to Q1, Q2 did not reach statistical significance, while Q3 and Q4 showed significant associations (OR 2.58, 95% CI: 1.65, 4.04; OR 6.30, 95% CI: 3.63, 10.93). In Model 2, Q2 still did not reach statistical significance, while Q3 and Q4 showed significant differences (OR 2.19, 95% CI: 1.23, 3.88; OR 5.03, 95% CI: 2.39, 10.57). In Model 3, neither Q2 nor Q3 showed significant differences, while Q4 was significantly associated with the risk of gallstones (OR 3.40, 95% CI: 1.33, 8.71). The trend test results for the three models (P for trend) were <0.001, <0.001, and 0.005, respectively, indicating that as RFM increased, the risk of gallstones gradually increased. This trend was particularly evident in the RFM Q4 group, where the positive correlation with gallstone risk was more pronounced.

**Table 2:** Logistic regression analysis between RFM and gallstones prevalence.

**RFM**	**Model 1**	**Model 2**	**Model 3**
Q1	1.00 (Reference)	1.00 (Reference)	1.00 (Reference)
Q2	1.40 (0.76, 2.55)	1.26 (0.67, 2.36)	1.08 (0.51, 2.26)
Q3	2.58 (1.65, 4.04)	2.19 (1.23, 3.88)	1.72 (0.85, 3.45)
Q4	6.30 (3.63, 10.93)	5.03 (2.39, 10.57)	3.40 (1.33, 8.71)
*P* for trend	<0.001	<0.001	0.005

**Figure 2:**
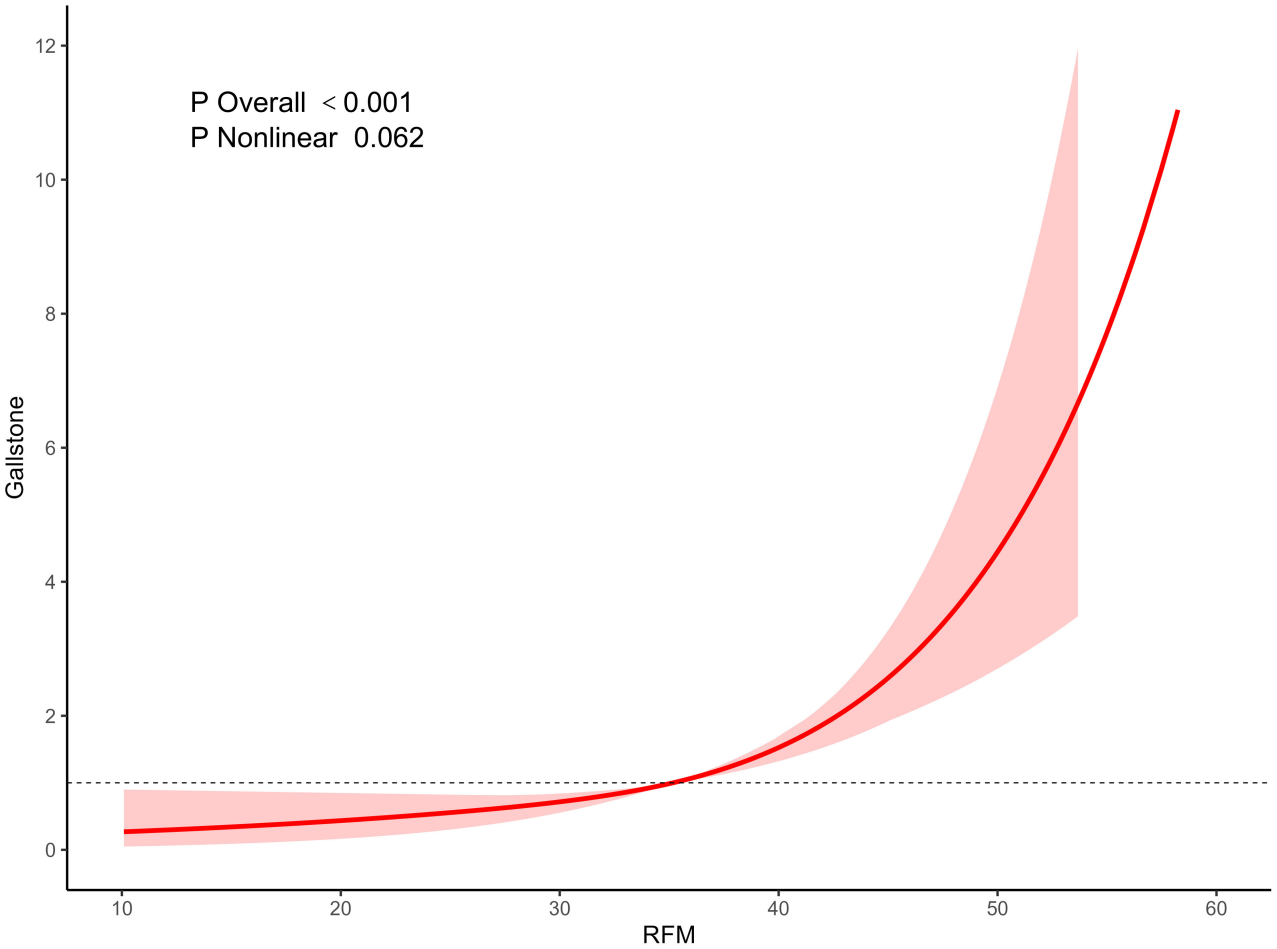
Association between RFM and gallstone using restricted cubic spline.

Further RCS regression analysis revealed that as RFM levels increased, the incidence of gallstones exhibited a gradual upward trend, especially in the RFM Q4 group, where the increase was particularly significant. The RCS curve illustrated a stepwise increase in the relationship between RFM and gallstone incidence, with the overall association test yielding a *P* < 0.001, thereby affirming a robust statistical relationship between RFM and gallstones. However, the *P*-value for the nonlinear association test was 0.062, implying that the correlation between RFM and gallstones may follow a linear trajectory (Figure 2).

### Subgroup analysis

The subgroup analysis revealed significant variations in the effect of RFM on gallstone risk across different populations. In the age subgroup, RFM in the <60 years group exhibited a significant positive association in the Q4 group (OR: 4.29, 95%CI: 1.29–14.22), whereas the ≥60 years group did not demonstrate a significant effect. In the gender subgroup, RFM in the female group showed significance in the Q4 group (OR: 13.30, 95%CI: 1.49- 118.54), while the male group did not exhibit a significant association. In the racial subgroup, RFM exhibited significant positive associations in both non-Hispanic Black (OR: 5.36, 95%CI: 1.97–14.59), non-Hispanic White groups (OR: 3.26, 95%CI: 1.19–8.99) and Other Race (OR: 8.32, 95%CI: 1.46–47.42), with notable differences observed in the Q4 group. In the education level subgroup, the moderate education level group displayed significant effects in both Q3 (OR: 2.47, 95%CI: 1.20–5.10) and Q4 groups (OR: 4.70, 95%CI: 1.86–11.91), while low and high education level groups did not show significant effects. The marital status analysis revealed a significant positive relationship in the Q4 group for the unmarried group (OR: 14.20, 95%CI: 3.64–55.42), whereas the cohabiting and single groups did not exhibit significant differences. In the subgroup with hypertension, RFM showed a significant positive relationship in the Q4 group, whereas no significant effects were observed in the subgroup without hypertension. Similarly, in the subgroup without diabetes and without hypercholesterolemia, RFM showed a significant positive relationship, while no significant effects were found in the diabetes or hypercholesterolemia subgroups. Smokers and moderate drinkers also exhibited significant positive associations in the Q4 group (OR: 3.89, 95%CI: 1.24–12.19; OR: 4.73, 95%CI: 1.30–17.17). In this subgroup analysis, the interaction *P*-value results revealed a significant interaction between RFM and age, as well as hypercholesterolemia (*P* < 0.05), while no significant interactions were identified in other subgroup analyses (Table 3).

**Table 3:** Subgroup analysis of the association between RFM and gallstone prevalence.

**Subgroup**	**RFM**				***P* for interaction**
	**Q1**	**Q2**	**Q3**	**Q4**	
**Age**					0.020
<60 years	Ref	0.80 (0.32,2.03)	2.27 (0.86,6.02)	4.29 (1.29,14.22)	
≥60 years	Ref	1.28 (0.48,3.40)	0.92 (0.30,2.78)	1.85 (0.42,8.08)	
**Gender**					0.221
Male	Ref	1.02 (0.47,2.23)	1.33 (0.56,3.15)	0.00 (0.00,0.00)^a^	
Female	Ref	3.28 (0.23,47.29)	7.02 (0.74.66.93)	13.30 (1.49,118.54)	
**Race**					0.149
Mexican American	Ref	3.81 (0.12,118.20)	4.27 (0.31,58.95)	7.36 (0.46,118.37)	
Non-Hispanic Black	Ref	1.22 (0.38,3.99)	1.80 (0.71,4.60)	5.36 (1.97,14.59)	
Non-Hispanic White	Ref	0.91 (0.39,2.11)	1.75 (0.84,3.66)	3.26 (1.19, 8.99)	
Other Hispanic	Ref	0.55 (0.21,1.40)	0.85 (0.35,2.06)	1.53 (0.56, 4.18)	
Other race	Ref	3.78 (1.09,13.05)	2.34 (0.54,10.08)	8.32 (1.46,47.42)	
**Education category**					0.243
Low	Ref	0.94 (0.23,3.81)	1.91 (0.57,6.41)	4.48 (0.94,21.24)	
Moderate	Ref	1.34 (0.55,3.28)	2.47 (1.20,5.10)	4.70 (1.86,11.91)	
High	Ref	0.80 (0.31,2.05)	0.93 (0.33,2.61)	1.85 (0.51,6.70)	
**Marital**					0.279
Cohabitation	Ref	0.85 (0.37,1.96)	1.46 (0.62,3.45)	2.63 (0.85,8.12)	
Never married	Ref	1.62 (0.40,6.62)	2.80 (0.92,8.55)	14.20 (3.64,55.42)	
Living alone	Ref	2.48 (0.75,8.14)	2.76 (0.78,9.76)	4.42 (0.92, 21.20)	
**Hypertension**					0.558
No	Ref	1.04 (0.36,2.98)	1.81 (0.65,5.03)	3.15 (0.95,10.42)	
Yes	Ref	0.98 (0.42,2.30)	1.41 (0.54,3.69)	3.67 (1.26,10.68)	
**Diabetes** ^b^					0.616
No	Ref	1.02 (0.48,2.16)	1.68 (0.75,3.79)	3.29 (1,23, 8.84)	
Yes	Ref	1.12 (0.27,4.69)	1.56 (0.28,8.73)	3.58 (0.52,24.64)	
**Cholesterol**					0.031
No	Ref	1.45 (0.54,3.94)	2.08 (0.77,5.61)	6.53 (1.91,22.37)	
Yes	Ref	0.76 (0.32,1.83)	1.20 (0.43,3.32)	1.39 (0.49,3.95)	
**Smoke**					0.058
No	Ref	0.83 (0.31,2.21)	1.11 (0.46,2.68)	2.96 (0.99,8.80)	
Yes	Ref	1.38 (0.60,3.15)	2.59 (1.00,6.68)	3.89 (1.24,12.19)	
**Alcohol group**					0.108
Low	Ref	1.23 (0.36,4.24)	1.95 (0.63,6.05)	3.51 (0.98,12.64)	
Moderate	Ref	0.73 (0.24,2.17)	1.24 (0.41,3.72)	4.73 (1.30,17.17)	
High	Ref	1.37 (0.41,4.63)	1.99 (0.34,11.70)	1.80 (0.28,11.66)	

All variables were included in the adjustment for subgroup analysis, except for the effect modifier.

a There are only 2 individuals in the male subgroup of the RFM Q4 category, making the comparison meaningless.

b The sample size in the borderline diabetes subgroup is too small to make a meaningful comparison.

### Predictive performance

**Figure 3:**
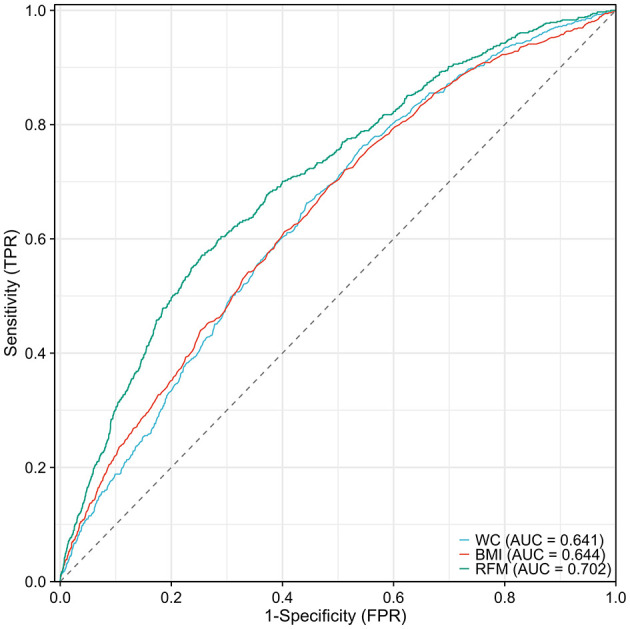
Comparison of Predictive Performance of RFM, WC, and BMI for Gallstones Using ROC Curve Analysis.

The study further compared the predictive performance of RFM, WC, and BMI for gallstones using ROC curve analysis. The results demonstrated that the AUC value for WC was 0.641 (95% CI: 0.621–0.661), for BMI it was 0.644 (95% CI: 0.623–0.664), whereas the AUC value for RFM was 0.702 (95% CI: 0.682–0.722). The DeLong's test showed that the diagnostic performance of WC and BMI in predicting gallstone was not statistically significant (*p* > 0.05). However, RFM demonstrated statistically significant diagnostic performance in predicting gallstones, compared to both WC and BMI (*p* < 0.05). These results suggest that RFM possesses higher discriminative ability in predicting gallstone risk, with its predictive efficacy being more prominent compared to WC and BMI (Figure 3).

## Discussion

Gallstone is a common digestive disorder influenced by complex metabolic and lifestyle factors, with obesity being a well-established risk factor. This study, utilizing data from the NHANES, systematically evaluates the relationship between RFM and gallstone formation. The findings include: (1) A significant positive correlation between RFM and gallstone prevalence, particularly in the highest RFM quartile (Q4), where the risk of gallstones was notably increased (OR = 3.40, 95% CI: 1.33–8.71); (2) RFM demonstrated superior predictive ability for gallstones compared to traditional BMI and WC, with an AUC of 0.702, significantly higher than BMI (AUC = 0.644) and WC (AUC = 0.641); (3) Subgroup analyses revealed significant variations in the impact of RFM on gallstone risk across different populations, particularly among individuals under 60 years, females, and non-Hispanic Black and White populations.

Obesity significantly increases the risk of gallstone formation by altering bile composition and gallbladder function (20, 21). Obese individuals often have elevated cholesterol levels in bile, which can exceed its solubilizing capacity, promoting the formation of cholesterol stones (22, 23). Moreover, obesity impacts fat metabolism and hormone regulation, including insulin resistance and elevated fatty acids, impairing gallbladder motility and leading to bile stasis, which further contributes to cholesterol deposition. Abdominal obesity, in particular, is strongly associated with a higher risk due to its effect on lipid metabolism and endocrine function (24). In addition to these factors, the positive correlation between RFM and gallstone prevalence highlights the role of visceral fat in gallstone formation. RFM more accurately reflects visceral fat accumulation, which is known to release free fatty acids (FFAs) and inflammatory cytokines. These disrupt lipid metabolism, causing cholesterol supersaturation in bile and increasing the risk of gallstones (25, 26). Furthermore, visceral fat is closely linked to insulin resistance, which impairs gallbladder motility and bile emptying, thereby further facilitating gallstone formation (27, 28). In addition to these metabolic and lipid-related factors, alterations in gastrointestinal hormone secretion, such as GLP-1, PYY, and ghrelin, in obesity and metabolic syndrome also contribute to the complex mechanisms leading to cholesterol supersaturation. These hormonal changes affect satiety, insulin secretion, and gut motility, exacerbating the metabolic disturbances associated with gallstone formation. Recent studies suggest that these alterations play a role in the pathophysiology of gallstones, particularly in the context of weight regain after bariatric surgery (29).

The superior predictive performance of RFM over BMI and WC may arise from its incorporation of both height and WC, offering a more comprehensive assessment of body fat distribution. In contrast, BMI does not differentiate between fat and muscle mass, while WC, although reflective of abdominal fat, does not account for the impact of height on fat distribution (8, 9). Therefore, RFM, as a composite measure, provides a more accurate prediction of gallstone risk (30). Considering the potential challenges of overfitting, it is important to validate these findings in larger and more diverse populations to ensure that RFM consistently outperforms other metrics across different settings.

The findings of this study are consistent with the existing literature on the relationship between obesity and gallstones. Numerous studies have established obesity as a significant risk factor for gallstones, with a particular emphasis on the role of visceral fat accumulation (31–33). However, traditional obesity indicators, such as BMI and WC, have limitations in predicting gallstone risk. This study highlights RFM as a novel body fat measurement that offers superior predictive accuracy, in line with recent research on RFM's application in metabolic diseases. For example, Woolcott et al. demonstrated that RFM outperforms both BMI and WC in predicting the risk of diabetes and cardiovascular disease (10).

Nevertheless, discrepancies exist, with some studies reporting stronger associations between BMI and gallstone risk (3), potentially due to differences in study populations and sample sizes. The strength of this study lies in its use of the large-scale NHANES database, which enables a more comprehensive evaluation of the RFM-gallstone relationship. Furthermore, subgroup analyses revealed variations in RFM's predictive utility across diverse populations, providing new insights for future precision medicine research.

In addition to RFM, this study explored other potential factors influencing gallstone formation. The results indicated that age, sex, race, education level, marital status, hypertension, diabetes, hypercholesterolemia, smoking, and alcohol consumption were all associated with gallstone prevalence. Metabolic disorders, including hypertension, diabetes, and hypercholesterolemia, may exacerbate gallstone risk by altering lipid metabolism and bile composition (5). Moreover, unhealthy lifestyle habits, such as smoking and alcohol consumption, may impair gallbladder function and bile secretion, thereby further increasing gallstone incidence (34).

Notably, subgroup analyses revealed significant variations in the impact of RFM on gallstone risk across diverse populations. For instance, the positive correlation between RFM and gallstones was more pronounced among younger individuals (<60 years) and females, potentially due to metabolic and hormonal differences. Future research should further explore these underlying mechanisms, particularly the role of hormones, such as estrogen, in gallstone formation.

Although there is no unified RFM threshold to predict gallstone formation, higher RFM values, reflecting visceral fat accumulation, may increase the risk of gallstones. In clinical practice, doctors should assess gallstone risk by combining RFM with other metabolic indicators (such as insulin resistance and obesity) and consider ultrasound screening for high-risk patients. Early detection of gallstones can help prevent complications. Future research should further explore the relationship between RFM and gallstones to provide clearer clinical guidelines.

## Limitations

While this study provides robust evidence for the RFM-gallstone relationship, it has several limitations. First, we acknowledge that using a questionnaire instead of ultrasound for gallstone diagnosis may limit the accuracy, potentially affecting the reliability of the study's findings. Second, its cross-sectional design precludes the ability to make causal inferences and does not eliminate the possibility of reverse causality (e.g., gallstones may induce metabolic disturbances that affect RFM). Third, although the NHANES database provides a large sample size, there may be potential selection bias. Despite multi-stage sampling and weighting adjustments, underrepresentation of certain groups (e.g., low-income or remote populations) may affect external validity. Non-response bias also remains a concern, as some health data may be incomplete. Additionally, RFM's reliance on height and weight measurements may introduce errors, potentially influencing fat mass estimation and gallstone risk prediction. Future studies should validate these findings across diverse populations. Furthermore, this study did not consider dietary factors, physical activity, socioeconomic status, liver function indicators, hematological disorders, cholesterol and triglycerides, genetic predisposition, and bypass surgeries, all of which may interact with RFM to influence gallstone risk. Future research should further investigate these interactions. While sensitivity analyses, such as penalized regression (e.g., Lasso) and bootstrapping, are important for assessing the robustness of findings, these analyses were not included in the current study. We recommend that future research incorporate these methods to further validate the reliability and generalizability of the observed results, particularly in smaller or imbalanced subgroups, to ensure the robustness of the conclusions drawn from this study. Future research should focus on validating RFM's applicability across diverse populations, exploring its relationship with other metabolic disorders, and establishing causal links to gallstones through longitudinal studies.

## Conclusion

This study, using the NHANES database, systematically explores the relationship between RFM and gallstone formation. The findings demonstrate a significant positive correlation between RFM and gallstone prevalence, especially in individuals with higher RFM levels. Furthermore, RFM outperformed traditional BMI and WC in predicting gallstone risk. Subgroup analyses revealed significant variations in the predictive utility of RFM across diverse populations. These findings offer new insights into the early prevention and intervention of gallstones. As a simple and accurate body fat measurement, RFM holds significant clinical value, particularly in screening and risk assessment for high-risk populations. In clinical practice, RFM could be employed as a valuable screening tool to identify individuals at higher risk for gallstones, especially in settings where more sophisticated diagnostic techniques are unavailable. By incorporating RFM measurements into routine clinical assessments, healthcare providers can better stratify patients based on their fat distribution, leading to more targeted preventive measures. While this cross-sectional study highlights the association between RFM and gallstone prevalence, causal relationships cannot be established. Longitudinal studies are needed to confirm the temporal link between RFM and gallstones and to validate its clinical utility for early risk identification.

## Data Availability

Publicly available datasets were analyzed in this study. The data used in this study are publicly available from NHANES database at https://www.cdc.gov/nchs/nhanes/index.htm.
